# Technology and myopia

**Published:** 2019-05-13

**Authors:** Pavan Kumar Verkicharla, Anthony Vipin Das

**Affiliations:** 1 Scientist: Prof Brien Holden Eye Research Centre, Brien Holden Institute of Optometry and Vision Sciences, L V Prasad Eye Institute, Hyderabad, India; 2 Associate Director & Consultant Ophthalmologist: Department of eyeSmart EMR & AEye, L V Prasad Eye Institute, Hyderabad, India.

**From mobile diagnostic devices to electronic medical record (EMR) systems, digitisation and artificial intelligence in eye care are fast evolving in South Asia.**

**Figure:**
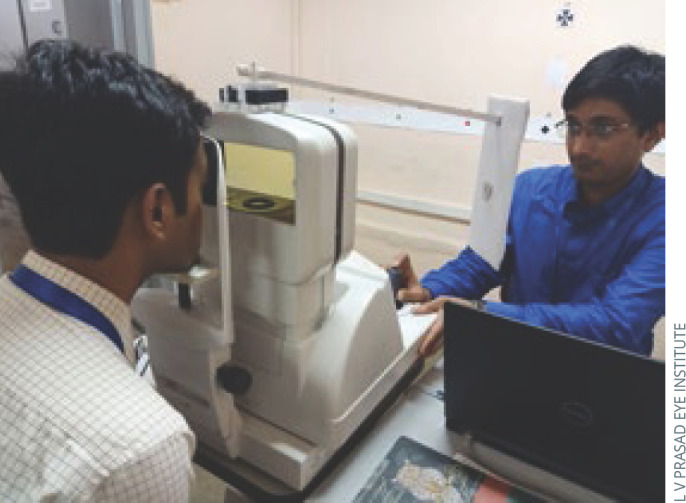
Open-field auto-refractor to determine peripheral refraction. INDIA

The use of technology has become integral to healthcare delivery globally. From mobile diagnostic devices for identifying causes of visual impairment (VI) to the electronic medical record (EMR) systems, digitisation is fast evolving in South Asia.

The EMR system can collect large datasets (“big data”) that are characterised by the four ‘V's - volume, variety, velocity and veracity.^1^ Refractive error data conform to all of the four criteria. Uncorrected refractive error is a major cause of visual impairment globally. The use of large datasets has the potential to understand the natural history of myopia at a population level. The advent of cloud technology enables aggregation, analysis and application of machine learning (ML) algorithms on large datasets from many hospitals.

Big data and machine learning models will help identify those children at risk of developing high myopia/ pathological myopia. However, the true potential of big data is still to be unlocked. Researchers at the LV Prasad Eye Institute are busy developing a machine learning model that will be able to predict the amount of myopia or refractive error progression in people under 25 years of age over a period of two years following their first visit.

The summary of artificial intelligence pipeline is given in Figure 1. The first step of the process is digitisation through EMR systems that includes variables such as age, gender, visual acuity, ocular diagnosis, and refractive error i.e. sphere, cylinder and axis for the prediction of myopia progression. The dataset is analysed with a machine learning (ML) model using gradient boosted tree regression, which is integrated into the EMR system through the cloud. Clinical validation of the ML model for prediction of myopia progression within an error range of 0.3 D is currently on-going.

**Figure 1:**
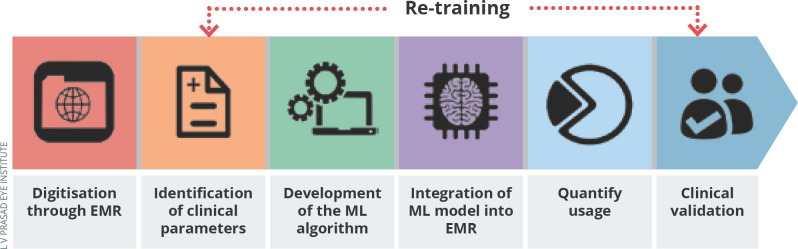
AEye pipeline for the application of machine learning models.

## Recent advancements in instrumentation

Research studies indicate that the risk factors for myopia can be classified into the following categories:

- genetic (both parents with myopia),
- optical (relative peripheral hyperopic refraction),
- structural (choroid, sclera in periphery, distorted or steeper retinal shape) and
- environmental factors (time spent outdoors and light exposure)^2^

Most of the instruments that determine either optical or the structural changes in the eye are designed to measure only the on-axis parameters. There is some evidence^3^ showing the importance of the peripheral retina in the genesis of myopia and thus triggering for modifications/customisations to the existing commercial systems to counter myopia progression based on measurements from peripheral retina. “Open-field” auto-refractors, unlike the regular auto-refractors, enable the fixation target to be placed in peripheral locations in the visual field to determine peripheral refraction. This technology has been used to assess peripheral refraction up to 30 degrees along horizontal and vertical meridian.^4^

**Figure:**
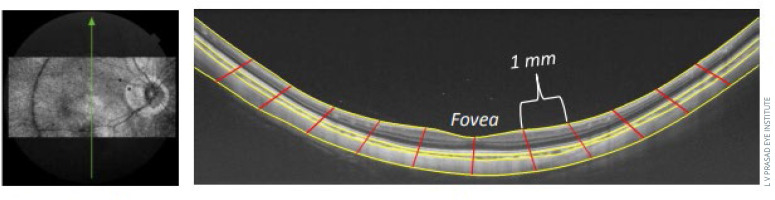
En-face image of retina (left) and segmented B-scan image of posterior ocular coats i.e. retina, choroid and sclera at different eccentricities (right). INDIA

Imaging algorithms have been developed using optical coherence tomography (OCT) to determine choroidal thickness and scleral thickness in different eccentricities along different meridians. Research is underway to identify any early signs in the periphery of the eye that can act as a marker for myopia, high myopia or pathologic myopia.

With regards to the environmental factors, in the last few decades, children were found to spend more time indoors with electronic gadgets and less time outdoors. Recent evidence from animal models and human studies indicate that time spent outdoors could be a modifiable risk factor for myopia development. This has led to development of wearable light sensing devices to quantify the amount of time spent outdoors and motivate children to increase this time.^5^

Emerging technologies that quantify risk factors for myopia present an opportunity to understand myopia progression and management. Customised open-field auto-refractors, state-of-the-art OCT image processing and machine learning algorithms can create a platform for characterising myopia and help in accurate prediction of its progression.

**Figure:**
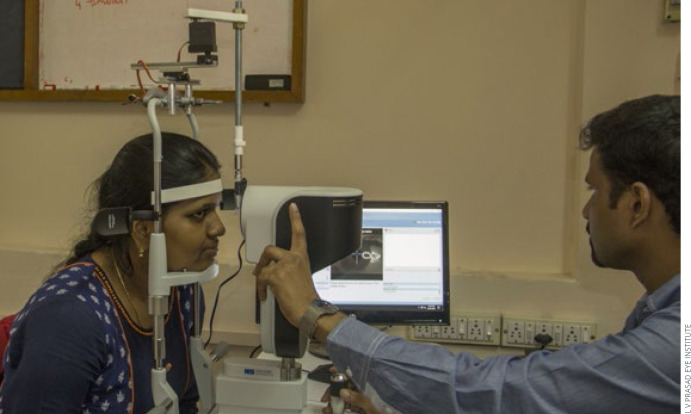
Instrument setup for peripheral eye length measurements and eye shape. INDIA
